# Supplementary material for: MUC1-C Dependence for the Progression of Pancreatic Neuroendocrine Tumors Identifies a Druggable Target for the Treatment of This Rare Cancer
Source: Biomedicines. 2024 Jul 8;12(7):1509. doi: 10.3390/biomedicines12071509 (PMC11274714; doi:10.3390/biomedicines12071509)
Supplement: Supplementary file 1 [file biomedicines-12-01509-s001.zip › biomedicines-3048901 - Supplementary file (2).pdf]

Supplemental Figure

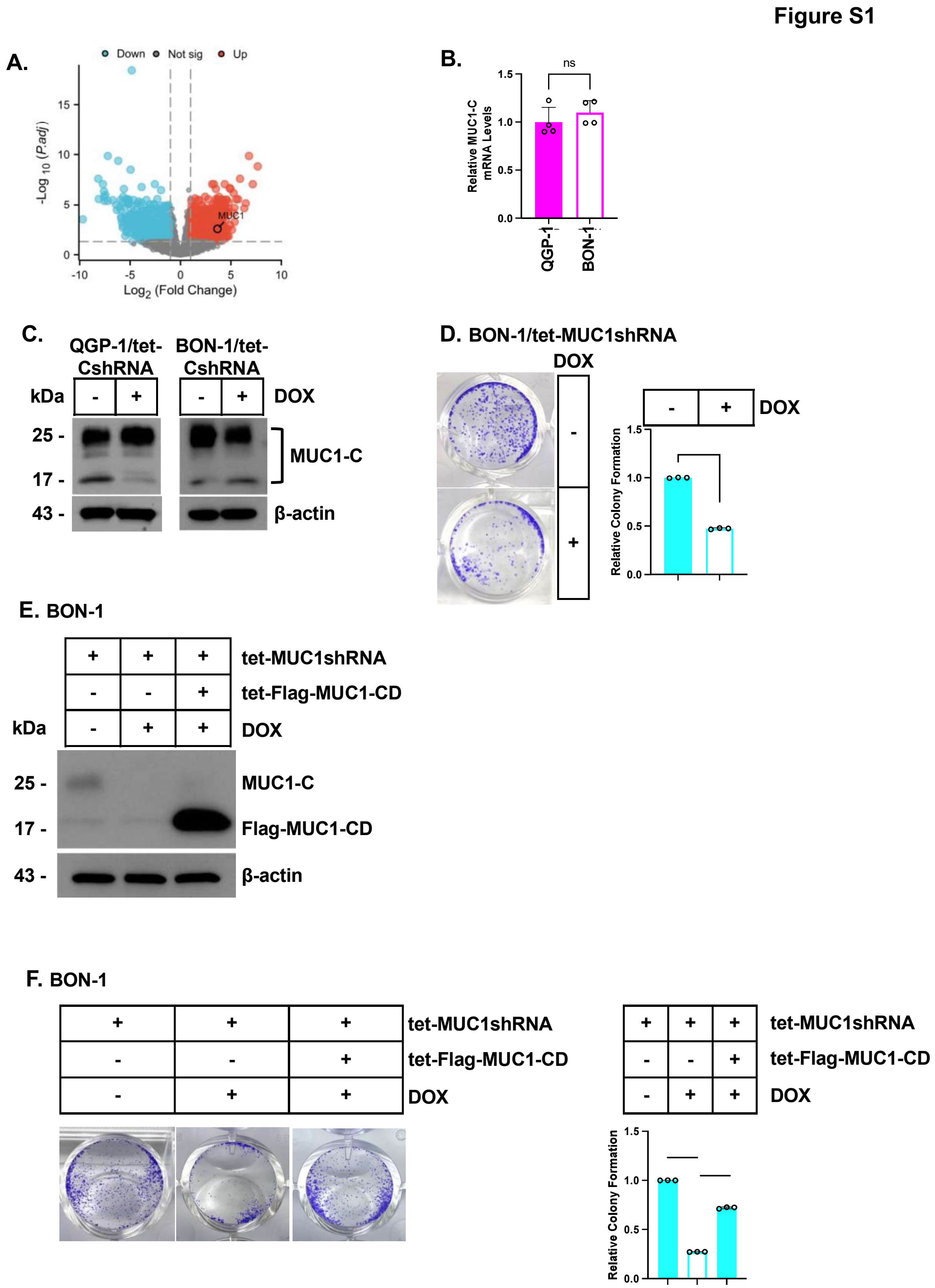

Figure S2

A. **QGP-1/tet-MUC1shRNA**

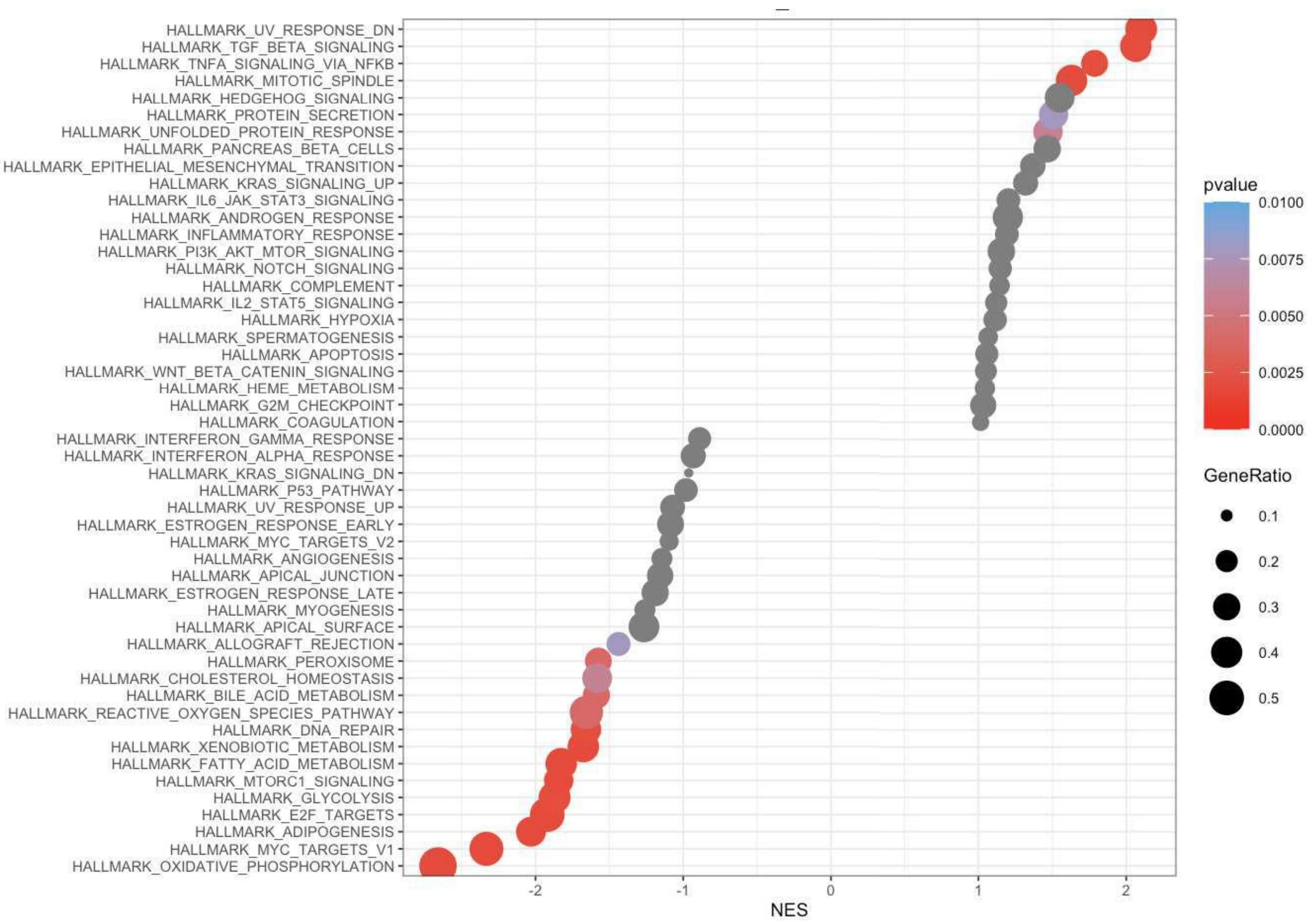

B. **BON-1/tet-MUC1shRNA**

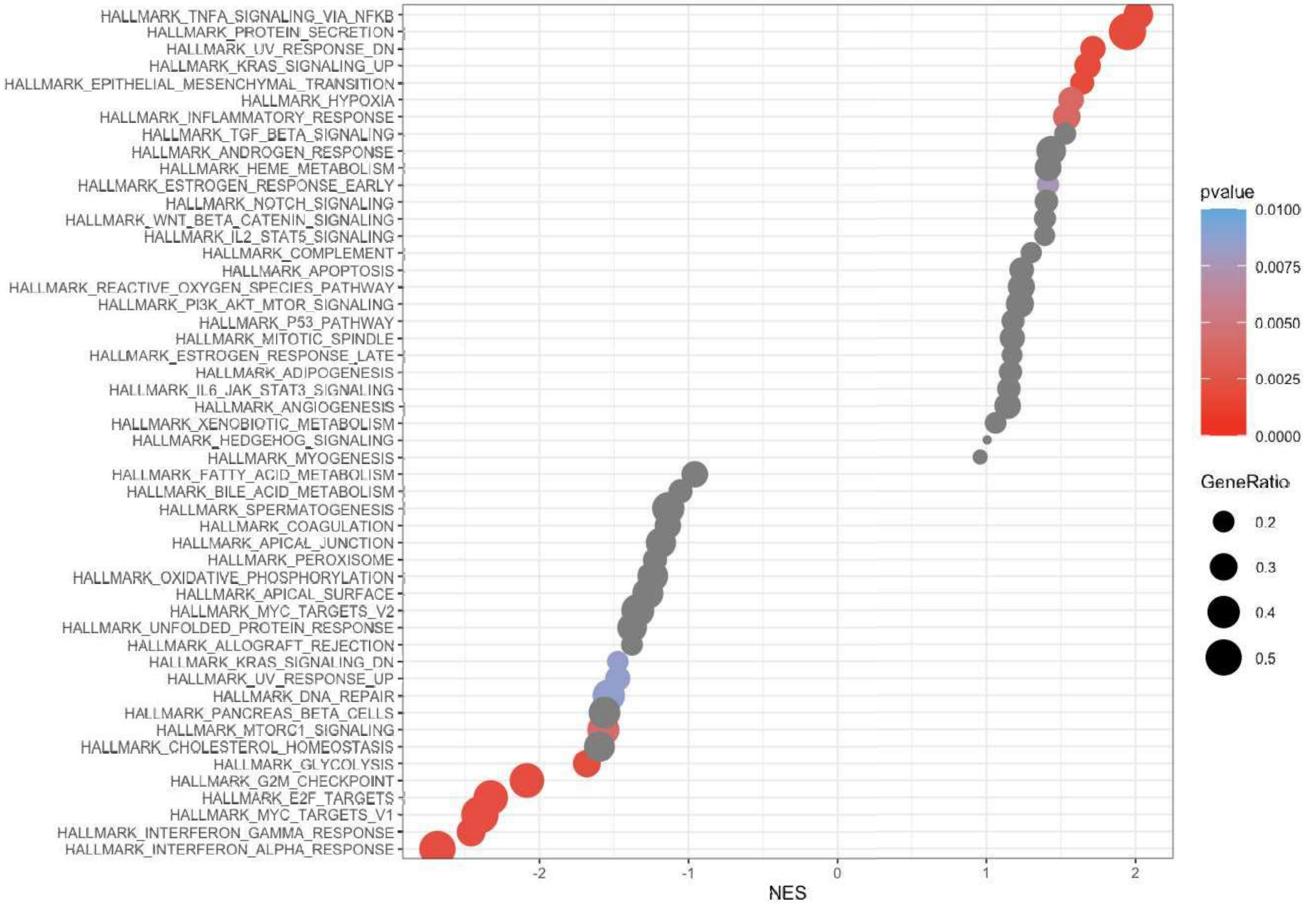

Figure S3

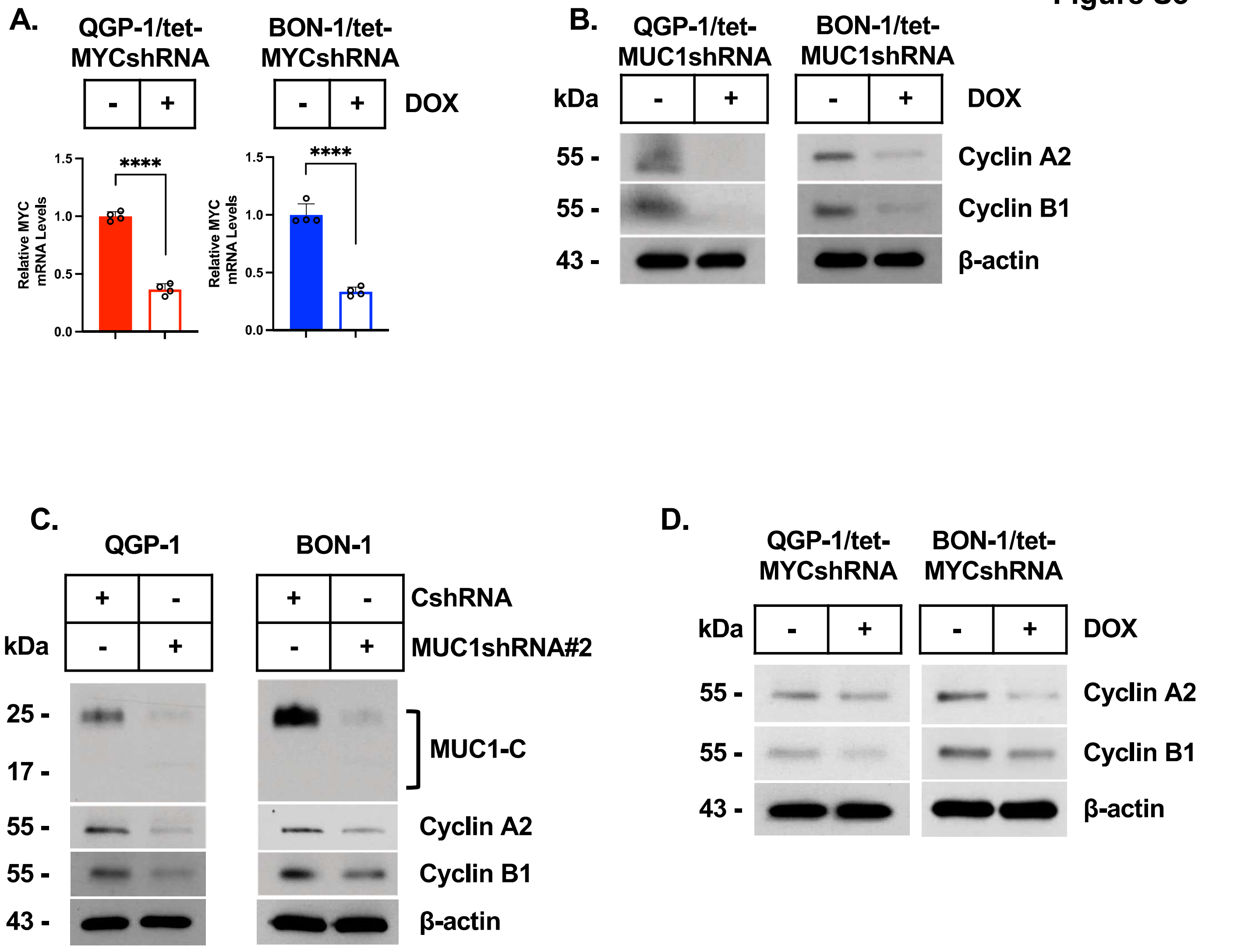
A.
QGP-1/tet-MYCshRNA
BON-1/tet-MYCshRNA
- + DOX
Relative MYC mRNA Levels
1.5
1.0
0.5
0.0
****
B.
QGP-1/tet-MUC1shRNA
BON-1/tet-MUC1shRNA
kDa
- + DOX
55 - Cyclin A2
55 - Cyclin B1
43 - β-actin
C.
QGP-1
BON-1
+ - CshRNA
- + MUC1shRNA#2
kDa
25 -
17 -
MUC1-C
55 - Cyclin A2
55 - Cyclin B1
43 - β-actin
D.
QGP-1/tet-MYCshRNA
BON-1/tet-MYCshRNA
kDa
- + DOX
55 - Cyclin A2
55 - Cyclin B1
43 - β-actin

Figure S4

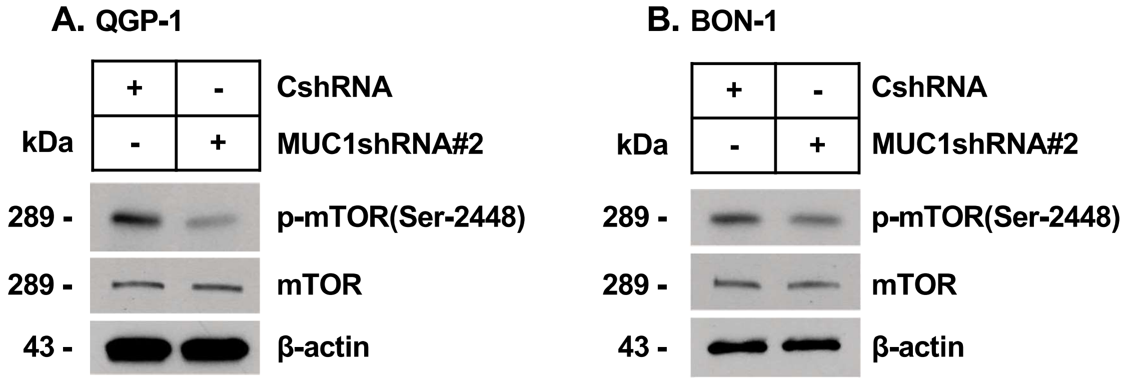
A. QGP-1
+
-
CshRNA
kDa
-
+
MUC1shRNA#2
289 -
p-mTOR(Ser-2448)
289 -
mTOR
43 -
β-actin
B. BON-1
+
-
CshRNA
kDa
-
+
MUC1shRNA#2
289 -
p-mTOR(Ser-2448)
289 -
mTOR
43 -
β-actin

Figure S5

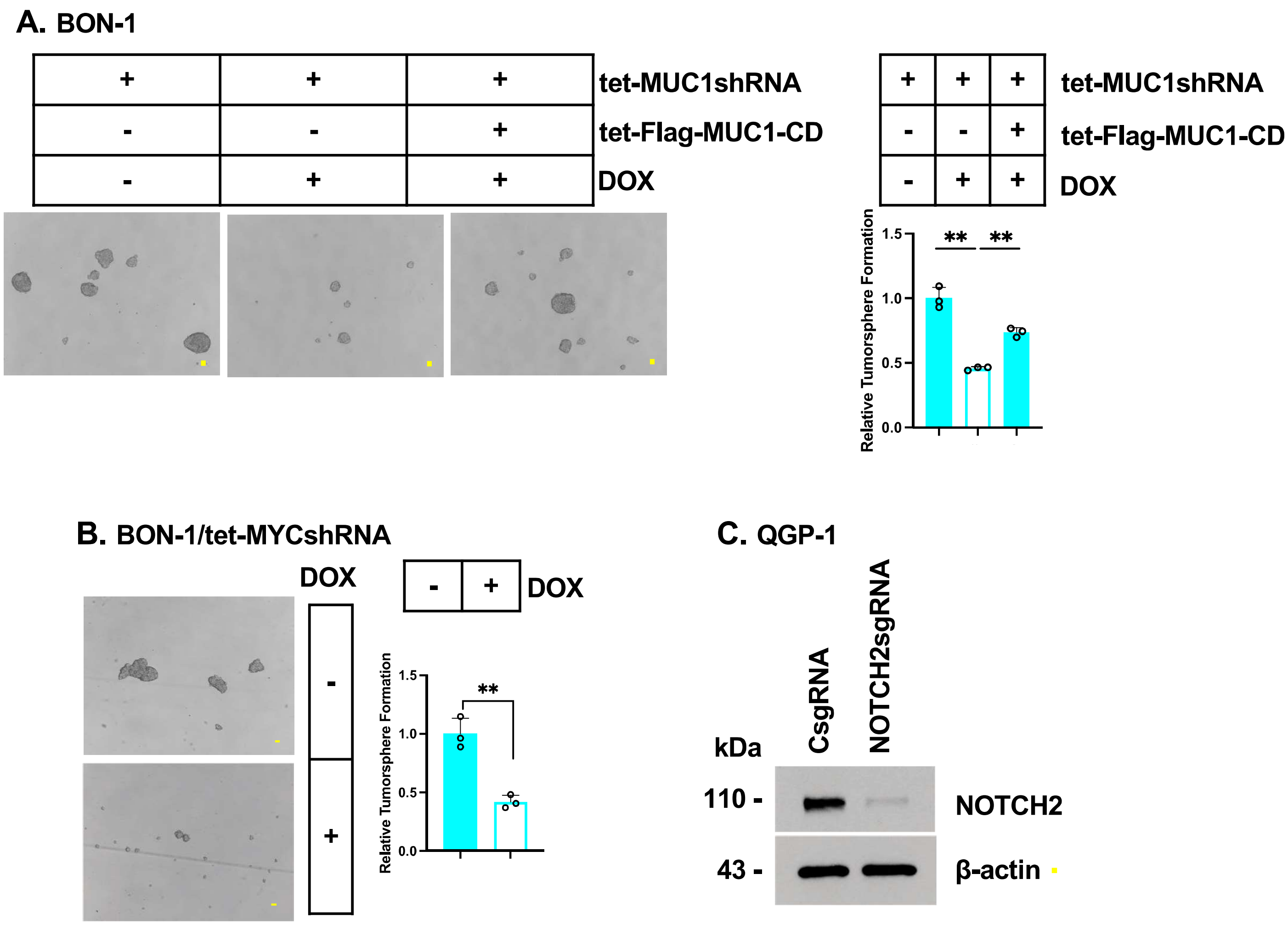

A. BON-1
+ + + tet-MUC1shRNA
- - + tet-Flag-MUC1-CD
- + + DOX
Relative Tumorsphere Formation
1.5
1.0
0.5
0.0
**
**
B. BON-1/tet-MYCshRNA
DOX
-
+
- + DOX
Relative Tumorsphere Formation
1.5
1.0
0.5
0.0
**
C. QGP-1
kDa
CsgRNA
NOTCH2sgRNA
110 -
NOTCH2
43 -
β-actin

Figure S6

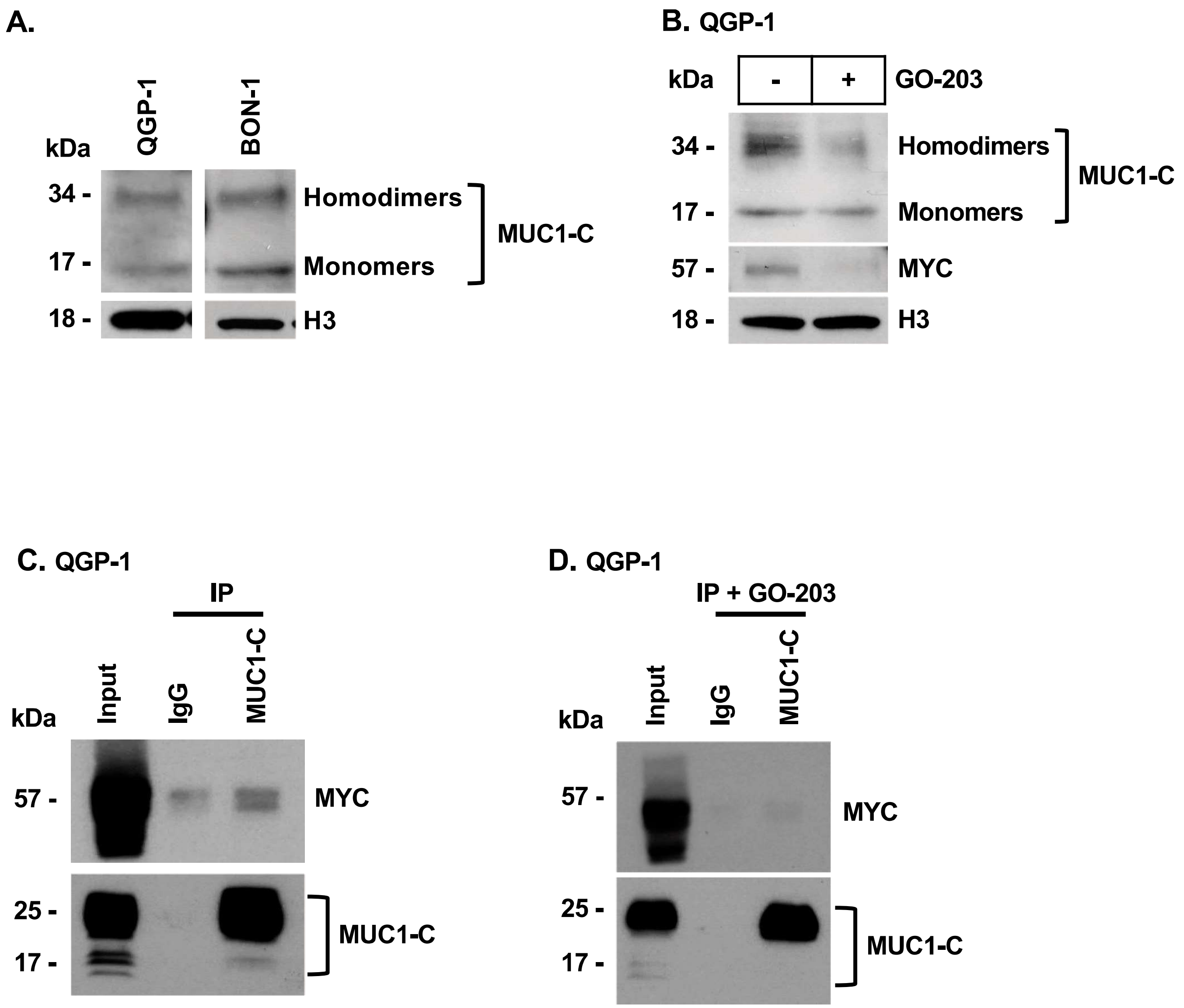
A.
QGP-1
BON-1
kDa
34 -
17 -
18 -
Homodimers
Monomers
MUC1-C
H3
B. QGP-1
kDa
-
+
GO-203
34 -
17 -
57 -
18 -
Homodimers
Monomers
MUC1-C
MYC
H3
C. QGP-1
IP
Input
IgG
MUC1-C
kDa
57 -
25 -
17 -
MYC
MUC1-C
D. QGP-1
IP + GO-203
Input
IgG
MUC1-C
kDa
57 -
25 -
17 -
MYC
MUC1-C

Figure S7

A.

Sample number	IHC image	Age	Sex	TNM classification	Location of metastasis	NET grade	DFS (Months)
1	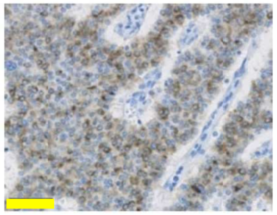	44	male	T3N1M0<br>Stage III	lymph node	G2	85
2	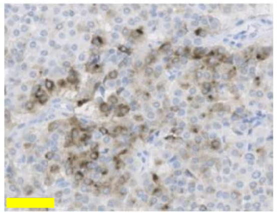	51	male	T3N1M1<br>Stage IV	liver<br>lymph node	G2	35
3	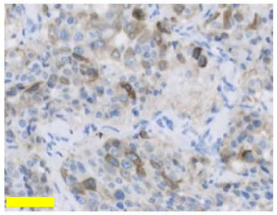	76	male	T3N1M0<br>Stage III	lymph node	G2	4
4	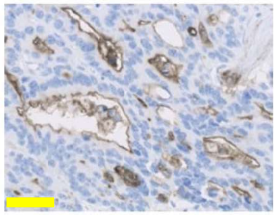	71	female	T3N0M0<br>Stege IIA	NA	G3	27
5	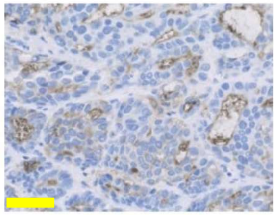	59	male	T3N0M0<br>Stage IIA	NA	G3	5

Supplemental Figure Legends

Supplemental Figure S1. Dependence of pNET cells on MUC1-C for colony formation. A. Volcano plot of genes downregulated (left) and upregulated (right) in primary pNET tumors from patients with metastatic vs localized disease. **B.** QGP-1 and BON-1 cells were analyzed for MUC1-C transcripts by qRT-PCR using primers listed in Supplemental Table S1. The results (mean±SD of 4 determinations) are expressed as relative levels compared to that obtained for QGP-1 cells (assigned a value of 1). **C.** Lysates from QGP-1/tet-CshRNA and BON-1/tet-CshRNA cells treated with vehicle of DOX for 7 days were immunoblotted with antibodies against the indicated proteins. **D.** BON-1/tet-MUC1shRNA cells treated with vehicle or DOX for 7 days were analyzed for colony formation. Shown are representative photomicrographs of stained colonies (left). The results (mean±SD of three determinations) are expressed as relative colony formation compared to that for vehicle-treated cells (assigned a value of 1)(right). **E.** Lysates from BON-1 cells expressing the indicated vectors treated with vehicle or DOX for 7 days were immunoblotted with antibodies against the indicated proteins. **F.** BON-1 cells expressing the indicated vectors treated with vehicle or DOX for 7 days were analyzed for colony formation. Shown are representative photomicrographs of stained colonies (above). The results (mean±SD of three determinations) are expressed as relative colony formation compared to that for vehicle-treated cells (assigned a value of 1)(below).

Supplemental Figure S2. MUC1-C regulates HALLMARK gene signatures in pNET cells. A and B. Top regulated HALLMARK gene signatures in QGP-1 (**A**) and BON-1 (**B**) cells with MUC1-C silencing.

Supplemental Figure S3. MUC1-C regulates MYC in pNET cells. A. QGP-1/tet-MYCshRNA and BON-1/tet-MYC1shRNA cells treated with vehicle or DOX for 7 days were analyzed for MYC transcripts by qRT-PCR. The results (mean±SD of 4 determinations) are expressed as relative levels compared to that obtained for vehicle-treated cells (assigned a value of 1). **B.** Lysates from QGP-1/tet-MUC1shRNA and BON-1/tet-MUC1shRNA cells treated with vehicle or DOX for 7 days were immunoblotted with antibodies against the indicated proteins. **C.** Lysates from QGP-1 and BON-1 cells expressing a CshRNA or MUC1shRNA#2 were immunoblotted with antibodies against the indicated proteins. **D.** Lysates from QGP-1/tet-MYCshRNA and BON-1/tet-MYCshRNA cells treated with vehicle or DOX for 7 days were immunoblotted with antibodies against the indicated proteins.

Supplemental Figure S4. MUC1-C/MYC signaling regulates the mTOR pathway. A and B. Lysates from QGP-1 (**A**) and BON-1 (**B**) cells expressing a CshRNA or MUC1shRNA#2 were immunoblotted with antibodies against the indicated proteins.

Supplemental Figure 5. MUC1-C/MYC signaling integrates the NOTCH2 pathway and self-renewal capacity. A. Representative images of tumorspheres derived from the indicated BON-1 cells treated with vehicle or DOX for 7 days. Bar represents 100 microns. The number of tumorspheres is expressed as the mean ± SD of three determinations. **B.** Representative images of tumorspheres derived from BON-1/tet-MYCshRNA cells treated with vehicle or DOX for 7 days. Bar represents 100 microns. The results (mean±SD of three determinations) are expressed as relative sphere formation compared to that for vehicle-treated cells (assigned a value of 1). **C.** Lysates from QGP-1/CsgRNA and QGP-1/NOTCH2sgRNA cells were immunoblotted with antibodies against the indicated proteins.

Supplemental Figure S6. Effects of treating pNET cells with the GO-203 inhibitor. A. Chromatin purified from QGP-1 and BON-1 cells was immunoblotted with antibodies against the indicated proteins. **B.** Chromatin from QGP-1 cells treated with 3 μM GO-203 for 4 days was immunoblotted with antibodies against the indicated proteins. **C and D.** Lysates from QGP-1 cells were incubated with a control IgG or anti-MUC1-C in the absence (**C**) and presence of 3 μM GO-203 for 2 days (**D**). Input proteins and precipitates were immunoblotted with antibodies against the indicated proteins.

Supplemental Figure S7. Association of MUC1-C expression in pNET tumors with adverse clinical outcomes. A. IHC staining of primary pNET tissues with a MUC1-C score of 2. Bar represents 50 microns.

Supplemental Tables

Supplemental Table S1. Primers used for qRT-PCR analyses.

Primer	FWD	REV
MUC1-C	TACCGATCGTAGCCCCTATG	CTCACCAGCCCAAACAGG
MYC	AGTAGAAATACGGCTGCACC	TTCGGGTAGTGGAAAACCAG
HMGA1	AGCGAAGTGCCAACACCTAAG	TGGTGGTTTTCCGGGTCTTG
HMGB3	CCCAGAGGTCCCTGTCAATTT	CGATCATAGCGCACTTTATCTGC
AURKB	CAGTGGGACACCCGACATC	GTACACGTTTCCAAACTTGCC
BUB3	GGTTCTAACGAGTTCAAGCTGA	GGCACATCGTAGAGACGCAC
RAN	GGTGGTACTGGAAAAACGACC	CCCAAGGTGGCTACATACTTCT
β-actin	GATGAGATTGGCATGGCTTT	CACCTTCACCGTTCCAGTTT

Supplemental Table S2. Downregulated E2F target genes shared in QGP-1 and BON-1 cells with MUC1-C silencing.

**ASF1B**	**MCM6**	**SHMT1**
**AURKB**	**MELK**	**SNRPB**
**CKS1B**	**MYBL2**	**SPC24**
**DDX39A**	**NME1**	**TK1**
**DEPDC1**	**POLA2**	**TMPO**
**GINS3**	**PRDX4**	**TUBG1**
**H2AFZ**	**RAN**	**XRCC6**
**HMGA1**	**RFC2**	
**HMGB3**	**RFC3**	
**HMMR**	**RNASEH2A**	

Supplemental Table S3. Downregulated cell cycle genes shared in QGP-1 and BON-1 cells with MUC1-C silencing.

PLIN3	NUP43	PTMS	NUF2
CENPA	MATN2	SHTN1	RHNO1
MAN1A2	MCM6	SINHCAF	ZNF414
CDC42EP1	MUC1	RAD51	MASTL
EFHC1	NFIA	RAN	PKMYT1
CKS1B	TUBD1	RFC2	CENPL
CTSD	LARP7	RFC4	BUB3
DNA2	POLA1	SDC1	AURKB
DR1	VPS37C	NSUN3	MORF4L2
FANCD2	FANCI	GINS3	ADGRE5
FANCG	ABHD10	HSPA13	MELK
RPL13A	PRIM1	TFF3	TOMM70
KBTBD2	ASF1B	TMPO	
WDR62	PBK	TSG101	
MRPS18B	PDXP	ZMYM1	
HMGB3	SLC44A2	CEP44	
HMMR	SLC39A10	CHAF1B	
AGFG1	SELENON	CDC7	

Supplemental Table S4. Downregulated MYC target genes shared in QGP-1 and BON-1 cells with MUC1-C silencing.

AIMP2	HNRNPC	RFC4	TFDP1
AP3S1	LDHA	RPL18	UBA2
APEX1	MCM6	RPLP0	VDAC3
BUB3	MRPL23	RPS3	XRCC6
CCT3	MRPS18B	RUVBL2	YWHAE
CCT5	NME1	SERBP1	
CCT7	PHB2	SF3B3	
EIF3D	PRDX4	SNRPD1	
H2AFZ	PSMD8	SNRPD2	
HNRNPA1	RAN	SSBP1	

Supplemental Table S5. Downregulated MTORC1 signaling genes shared in QGP-1 and BON-1 cells with MUC1-C silencing.

**ALDOA**	**FADS1**	**PHGDH**
**ATP6V1D**	**FADS2**	**PSAT1**
**CACYBP**	**FAM129A**	**PSMB5**
**CCNG1**	**G6PD**	**SHMT2**
**CORO1A**	**GMPS**	**SLC1A5**
**CXCR4**	**GSR**	**SSR1**
**DDIT4**	**HMBS**	**TPI1**
**DDX39A**	**IFI30**	**TUBA4A**
**EBP**	**LDHA**	**TUBG1**
**ENO1**	**LTA4H**	**WARS**

Supplemental Table S6. Clinical characteristics of patients with pNET tumors.

Factors	Total<br>n = 58
Age, years (mean ± SD)	61.9 ± 14.2
Sex, male/female	34(59) / 24(41)
Tumor size (mm)	19.2 ± 18.7
Functional / Non-functional	13(22) / 45(78)
NET Grade, G1/G2/G3	31(54)/ 24(41)/ 3(5)
TNM classification	
T 1/2/3/4	37(66)/ 10(18)/ 9(16)/ 0(0)
N 0/1	50(86)/ 8(14)
M 0/1	56(96)/ 2(4)
Stage I/II/III/IV	44(75)/ 12(21)/ 0(0)/ 2(4)
Metastasis at surgery, yes/no	8(14)/ 50(86)
MUC1-C staining score, 0/1/2	48(84)/ 5(8)/ 5(8)

Data are expressed as n (%) unless otherwise specified
